# Seroprevalence of *Toxoplasma gondii* infection and associated risk factors in Huicholes in Mexico

**DOI:** 10.1186/1756-3305-7-301

**Published:** 2014-07-01

**Authors:** Cosme Alvarado-Esquivel, Sandy Janet Pacheco-Vega, Jesús Hernández-Tinoco, Luis Francisco Sánchez-Anguiano, Luis Omar Berumen-Segovia, Francisco Javier Imard Rodríguez-Acevedo, Isabel Beristain-García, Elizabeth Rábago-Sánchez, Oliver Liesenfeld, Federico Campillo-Ruiz, Oscar Alberto Güereca-García

**Affiliations:** 1 Biomedical Research Laboratory, Faculty of Medicine and Nutrition, Juárez University of Durango State, Avenida Universidad S/N, 34000 Durango, Dgo, Mexico; 2 Institute for Scientific Research “Dr. Roberto Rivera-Damm”, Juárez University of Durango State, Avenida Universidad S/N, 34000 Durango, Mexico; 3 Servicios de Salud de Durango, Cuauhtémoc 225 norte, 34000 Durango, Mexico; 4 Facultad de Enfermería y Obstetricia, Juárez University of Durango State, Cuauhtémoc 223 norte, 34000 Durango, Mexico; 5 Institute for Microbiology and Hygiene, Campus Benjamin Franklin, Charité Medical School, Hindenburgdamm 27, D-12203 Berlin, Germany; 6 Present address: Roche Molecular Diagnostics, Pleasanton, CA, USA

**Keywords:** *Toxoplasma gondii*, Seroprevalence, Huicholes, Cross-sectional study

## Abstract

**Background:**

Very little is known about the seroepidemiology of *Toxoplasma gondii* infection in ethnic groups in Mexico. Huicholes are an indigenous ethnic group living in a remote mountainous region in Mexico. We sought to determine the prevalence of anti-*Toxoplasma* IgG and IgM antibodies in Huicholes; and to determine the association of *Toxoplasma* seropositivity with socio-demographic, behavioral, and clinical characteristics of Huicholes.

**Methods:**

We performed a cross sectional survey in Huicholes from September 2013 to January 2014. A convenience sampling method was used. We investigated the prevalence of anti-*Toxoplasma* IgG and IgM antibodies in 214 Huicholes using enzyme-linked immunoassays. A standardized questionnaire was used to obtain the characteristics of the Huicholes. Bivariate and multivariate analyses were used to assess the association of *Toxoplasma* exposure and Huicholes’ characteristics.

**Results:**

Of the 214 Huicholes studied (mean age: 37.98 ± 15.80 years), 71 (33.2%) were positive for anti-*T. gondii* IgG antibodies and 47 (66.2%) of them were also positive for anti-*T. gondii* IgM antibodies. Seroprevalence of *T. gondii* infection did not vary with age, sex, or occupation. However, seroprevalence of anti-*T. gondii* IgM antibodies was significantly higher in female than in male Huicholes. Multivariate analysis of socio-demographic and behavioral characteristics showed that *T. gondii* exposure was associated with consumption of turkey meat (OR = 2.28; 95% CI: 1.16-4.46; *P* = 0.01). In addition, seroprevalence of *T. gondii* infection was significantly higher in Huicholes suffering from dizziness and memory impairment than those without such clinical characteristics.

**Conclusions:**

Our results demonstrate serological evidence of *T. gondii* exposure among Huicholes which may be impacting their health. Results of this first study of *T. gondii* infection in Huicholes may be useful for the design of optimal preventive measures against infection with *T. gondii*.

## Background

Infections with the parasite *Toxoplasma gondii* (*T. gondii*) occur worldwide [1] and affect about one third of humanity [2]. Although most infections with *T. gondii* are asymptomatic, some infected individuals may suffer from symptomatic pathological changes in the lymph nodes, eyes, and central nervous system [3]. In addition, pregnant women with primary infection with *T. gondii* may transmit the infection to the fetus leading to congenital disease [4]. Immunocompromised individuals infected with *T. gondii* may develop a life-threatening disease [5]. Ingestion of food or water contaminated with oocysts shed by cats [3,6] and eating undercooked or raw meat containing tissue cysts [3,7,8] are important routes of *T. gondii* transmission.

Very little is known about the epidemiology of *T. gondii* infection in ethnic groups in Mexico. We have previously studied the seroepidemiology of *T. gondii* infection in Mennonites [9] and Tepehuanos [10] in Durango, Mexico. To the best of our knowledge, there is not any report about the epidemiology of *T. gondii* infection in Huicholes (an indigenous ethnic group living in a remote mountainous region (Sierra Madre Occidental) in the western central Mexican states of Nayarit, Durango, Jalisco and Zacatecas. Life style in Huicholes differs from that in other rural population groups in Durango; they live in marked poverty with very poor housing and sanitary conditions. They have limited access to health care services, and Hospitals in their region do not have a number of laboratory tests for diagnosis of infectious diseases i.e., infection with *T. gondii*. It is important to study the epidemiology of *T. gondii* infection in Huicholes because they live in a climatic scenario that may favor *T. gondii* infection. Huicholes live in a warmer and more humid area than other population groups in the region. Environmental factors may contribute to a higher seroprevalence of *T. gondii* infection [1,11]. In general, the seroprevalence of *T. gondii* infection is higher in humid climates than in dry climates; and this is the case in humans [12-14] and animals [15-17]. Furthermore, Huicholes eat meat from wild animals that may be infected with *T. gondii*. Therefore, we sought to determine the seroprevalence of *T. gondii* exposure in Huicholes and the association of *Toxoplasma* seropositivity with socio-demographic, behavioral, and clinical characteristics of Huicholes.

## Methods

### Study design and study population

We performed a cross sectional survey in Huicholes in Mexico from September 2013 to January 2014. Huicholes were sampled in the locality of Huazamota in the municipality of El Mezquital in Durango State, Mexico. Huazamota (23°28´N 104°24´W) has an altitude of 600 meters above sea level, a warm-sub-humid climate, and a mean annual temperature of 19.2°C. The south region of El Mezquital municipality has a mean annual rainfall varying from 800 to 1000 mm. Other ethnic groups live in the mountainous region including Mexicaneros and Tepehuanos. Inclusion criteria for the study subjects were: 1) Huichol ethnicity (people who speak the Huichol language and identify themselves as Huicholes); 2) aged 14 years and older; and 3) that voluntarily accepted to participate.

### Sample size and sampling method

For calculation of the sample size, we used a reference seroprevalence of 22.4% [10] as expected frequency of the factor under study, 7,000 as the size of population from which the sample was selected, 16.9% as the least acceptable result, and a confidence level of 95%. The result of the calculation was 214 subjects. A convenience sampling method was used. Specifically, the authors approached Huicholes leaders for permission and support; each leader communicated and invited all people under his command; those who accepted the invitation gathered in a specific area to provide socio-demographic data and blood sample; 214 people who met the inclusion criteria were enrolled.

### Socio-demographic, clinical, and behavioral data

We used a standardized questionnaire to obtain the socio-demographic, clinical and behavioral characteristics of the Huicholes. Socio-demographic items were age, sex, birth place, residence, educational level, occupation, and socioeconomic status. Clinical data included the presence of underlying diseases, presence or history of lymphadenopathy, frequent presence of headache, dizziness, impairments of memory, reflexes, hearing, and vision, and a history of surgery, blood transfusion or transplants. Clinical data including impairments was self-reported. Huicholes were considered “ill” when they suffered from any disease either acute or chronic affecting any organ or system i.e. digestive, respiratory, circulatory, endocrine, or nervous, and included any psychiatric, rheumatic, hematological or nutritional disorder and any type of morbidity. In Huichol women, obstetric data were also obtained. Behavioral items included animal contacts, contact with cat excrement, foreign travel, meat consumption (pork, beef, goat, lamb, boar, chicken, turkey, pigeon, duck, rabbit, venison, squirrel, horse, opossum, or other), frequency of meat consumption, consumption of raw or undercooked meat, unpasteurized milk, dried or processed meat (ham, sausages or chorizo), consumption of unwashed raw vegetables, fruits, or untreated water, frequency of eating away from home (in restaurants or fast food outlets), contact with soil (gardening or agriculture), and type of flooring at home from all participants were obtained.

### Serological examination for *T. gondii* antibodies

Serum samples were obtained from about 3 ml of whole blood. Sera were kept frozen at −20°C until analyzed. All sera were analyzed by qualitative and quantitative methods for anti-*T. gondii* IgG antibodies with a commercially available enzyme immunoassay “*Toxoplasma* IgG” (Diagnostic Automation Inc., Calabasas, CA, USA). Anti-*T. gondii* IgG antibody levels were expressed as International Units (IU)/ml, and a cut-off of ≥ 8 IU/ml was used for seropositivity. In addition, sera positive for *T. gondii* IgG were further analyzed for anti-*T. gondii* IgM antibodies by a commercially available enzyme immunoassay “*Toxoplasma* IgM” kit (Diagnostic Automation Inc., Calabasas, CA, USA). The cut-off for IgM seropositivity for each assay was obtained by multiplying the mean cut-off calibrator optical density by a correction factor (f = 0.35-0.40) printed on the label of calibrator. All assays were performed following the instructions of the manufacturer and included positive and negative controls in each run. A positive IgG test and a negative IgM test in a participant was interpreted as a latent infection. A positive IgG test and a positive IgM test in a participant was interpreted as probability of a recent or acute infection.

### Statistical analysis

We used the Epi Info version 3.5.4 and SPSS version 15.0 software for the statistical analysis. The Pearson’s chi-square test and the Fisher exact test (when values were less than 5) were used for initial comparison of the frequencies among groups. Multivariate analysis was used to assess the association between the characteristics of the Huicholes and the seropositivity to *T. gondii*. Variables were included in the multivariate analysis if they had a *P* value equal to or less than 0.15 in the bivariate analysis. Odds ratio (OR) and 95% confidence interval (CI) were calculated by multivariate analysis using the Enter method. The Hosmer-Lemeshow goodness of fit test was used to assess the fitness of the regression model. A *P* value <0.05 was considered statistically significant.

### Ethical aspects

The purpose and procedures of the survey were explained to all Huicholes. This study was approved by the Ethical Committee of the General Hospital of the Secretary of Health in Durango City, Mexico. Participation in the study was voluntary and a written informed consent was obtained from all participants and from the next of kin of minor participants. All Huicholes were proficient in Spanish and understood explanations about the purpose and procedure of the survey as well as the informed consent provided by the interviewers. Results of the laboratory tests were sent to the Huicholes’ nearest Hospital (Huazamota) where health care providers could inform participants about their results and provide them with medical care if needed.

## Results

In total, we enrolled 214 Huicholes in the study including 86 (40.2%) males and 128 (59.8%) females. Most Huicholes were born in Durango; their mean age was 37.98 ± 15.80 years (range 14–82 years). General socio-demographic characteristics of the 214 Huicholes studied are shown in Table 1.

**Table 1:** **Socio-demographic characteristics of Huicholes and seroprevalence of *T. gondii *** **infection**

**Characteristic**	**No. of subjects tested**	**Prevalence of *T. gondii *** **infection**		***P *** **value**
		**No.**	**%**	
Age groups (years)				
30 or less	80	21	26.3	0.09
31-50	90	30	33.3	
>50	44	20	45.5	
Sex				
Male	86	29	33.7	0.89
Female	128	42	32.8	
Birth place				
Durango state	181	57	31.5	0.22
Other Mexican state	33	14	42.4	
Residence				
Durango state	210	69	32.9	0.6
Other Mexican State or abroad	4	2	50.0	
Educational level				
No education	87	35	40.2	0.02
1-6 years	77	27	35.1	
>6 years	50	9	18.0	
Occupation				
Labourer^a^	107	34	31.8	0.66
Non-labourer^b^	107	37	34.6	
Socio-economic level				
Low	201	69	34.3	0.16
Medium	13	2	15.4	

^a^Labourer: Agriculture, construction, business, livestock raising, factory worker, other.

^b^Non-labourer: student, housekeeping or none occupation.

Of the 214 Huicholes studied, 71 (33.2%) were positive for anti-*T. gondii* IgG antibodies and 47 (66.2%) of them had anti-*T. gondii* IgM antibodies. Of the 71 anti-*T. gondii* IgG positive participants, 26 (36.6%) had IgG levels higher than 150 IU/ml, 1 (1.4%) between 100 to 150 IU/ml, and 44 (62.0%) between 9 to 99 IU/ml.

Seroprevalence of *T. gondii* infection did not vary significantly with age, sex, birthplace, residence, occupation or socioeconomic level of Huicholes (Table 1). In contrast, seroprevalence varied significantly with educational level; Huicholes with no education had the highest seroprevalence of *T. gondii* exposure (40.2%). In the 71 Huicholes seropositive for anti-*T. gondii* IgG antibodies, the prevalence of anti-*T. gondii* IgM antibodies was significantly higher in female (33/42: 78.6%) than in male (14/29: 48.3%), Huicholes (*P* = 0.008). Prevalence of high (>150 IU/ml) IgG antibody levels was similar in female (16/128: 12.5%) and male (10/86: 11.6%), Huicholes (*P* = 0.84).

Concerning clinical characteristics, seroprevalence of anti-*T. gondii* IgG was significantly higher in Huicholes suffering from dizziness and memory impairment than those without such clinical characteristics (Table 2). The frequency of *T. gondii* exposure in subjects with dizziness and memory impairment did not vary with age (*P* = 0.19 and *P* = 0.48, respectively). The frequencies of other clinical characteristics including the presence of underlying diseases; suffering from frequent headaches; presence or history of lymphadenopathy; reflexes, hearing and visual impairments; histories of surgery, blood transfusion, and transplant were similar among *T. gondii* positive and *T. gondii* negative individuals. Histories of miscarriage and stillbirth in women were not associated with *T. gondii* seropositivity.

**Table 2:** **Bivariate analysis of clinical data and infection with *T. gondii *** **in Huicholes**

**Characteristic**	Subjects tested* no.	**Prevalence of *T. gondii *** **infection**		***P *** **value**
		**No.**	**%**	
Clinical status				
Healthy	171	59	34.5	0.34
Ill	41	11	26.8	
Lymphadenopathy ever				
Yes	71	28	39.4	0.17
No	143	43	30.1	
Headache frequently				
Yes	154	57	37	0.05
No	60	14	23.3	
Memory impairment				
Yes	123	48	39	0.03
No	91	23	25.3	
Dizziness				
Yes	123	51	41.5	0.004
No	85	19	22.4	
Reflexes impairment				
Yes	73	29	39.7	0.14
No	141	42	29.8	
Hearing impairment				
Yes	19	8	42.1	0.38
No	195	63	32.3	
Visual impairment				
Yes	56	24	42.9	0.07
No	158	47	29.7	
Surgery ever				
Yes	26	8	30.8	0.78
No	188	63	33.5	
Transplantation				
Yes	0	0	0	-
No	214	71	33.2	
Blood transfusion				
Yes	20	7	35	0.85
No	194	64	33	
Pregnancies				
None	11	0	0	0.07
One to three	40	13	32.5	
Four to six	44	15	34.1	
More than six	33	14	42.4	
Deliveries				
Zero	14	2	14.3	0.26
One to three	44	12	27.3	
Four to six	44	16	36.4	
Seven to nine	19	9	47.4	
Nine to twelve	7	3	42.9	
Cesarean sections				
No	115	37	32.2	0.64
Yes	13	5	38.5	
Miscarriages				
No	97	32	33	0.51
Yes	25	10	40	
Stillbirths				
No	115	37	32.2	0.22
Yes	7	4	57.1	

*Subjects with available data.

With respect to behavioral characteristics, a number of variables showed *P* values lower than 0.15 in the bivariate analysis including presence of dogs at home (*P* = 0.06), consumption of raw dried meat (*P* = 0.14), and consumption of meat from goat (*P* = 0.14), turkey (*P* = 0.002), and pigeon (*P* = 0.04). A selection of behavioral characteristics and their correlation with *T. gondii* exposure are shown in Table 3. Other behavioral characteristics of Huicholes including contact with cats, cleaning cat excrement, raising animals, traveling, consumption of meat other than goat, turkey and pigeon meat, frequency of meat consumption, degree of meat cooking, consumption of unpasteurized milk, processed meat, unwashed raw vegetables or fruits, untreated water, frequency of eating out of home, soil contact, and soil flooring at home showed *P* values higher than 0.15 in the bivariate analysis. Further analysis by using logistic regression showed that *T. gondii* exposure was only associated with consumption of turkey meat (OR = 2.28; 95% CI: 1.16-4.46; *P* = 0.01) (Table 4). A *P* = 0.35 was obtained in the Hosmer-Lemeshow test indicating an acceptable fit of our regression model.

**Table 3:** **Bivariate analysis of selected putative risk factors for infection with *T. gondii *** **in Huicholes**

**Characteristic**	Subjects tested* no.	**Prevalence of *T. gondii *** **infection**		***P *** **value**
		**No.**	**%**	
Cats at home				
Yes	93	33	35.5	0.53
No	121	38	31.4	
Dogs at home				
Yes	165	60	36.4	0.06
No	49	11	22.5	
Goat meat consumption				
Yes	193	67	34.7	0.14
No	21	4	19	
Chicken meat consumption				
Yes	209	71	34	0.17
No	5	0	0	
Turkey meat consumption				
Yes	106	46	43.4	0.002
No	108	25	23.1	
Pigeon meat consumption				
Yes	176	63	35.8	0.04
No	37	7	18.9	
Venison consumption				
Yes	203	69	34	0.34
No	11	2	18.2	
Squirrel meat consumption				
Yes	63	25	39.7	0.19
No	151	46	30.5	
Raw dried meat				
Yes	122	46	37.7	0.14
No	86	24	27.9	
Floor at home				
Ceramic or wood	6	3	50	0.19
Concrete	102	28	27.5	
Soil	106	40	37.7	

*Subjects with available data.

**Table 4:** **Multivariate analysis of selected characteristics of Huicholes and their association with *T. gondii *** **infection**

**Characteristic**	**Odds ratio**	**95% confidence interval**	***P *** **value**
Age (years)			
30 or less	1.00		
31-50	0.73	0.32-1.66	0.45
> 50	1.23	0.44-3.47	0.68
Educational level			
No education	2.08	0.83-5.19	0.11
1-6 years	1.92	0.68-5.38	0.21
> 6 years	1.00		
Contact with dogs	2.11	0.93-4.78	0.07
Consumption of:			
Goat meat	1.61	0.46-5.60	0.45
Turkey meat	2.28	1.16-4.46	0.01
Pigeon meat	1.35	0.51-3.55	0.53
Raw dried meat	1.20	0.63-2.30	0.56

In the 71 Huicholes with anti-*T. gondii* IgG antibodies, seroprevalence of anti-*T. gondii* IgM antibodies was higher (*P* = 0.006) in Huicholes who raised animals (41/55; 74.5%) than in those without such practice (6/16: 37.5%), and in Huicholes with consumption of raw dried meat (35/46; 76.1%) than in those without such eating habit (11/24: 45.8%) (*P* = 0.01).

## Discussion

The present study was performed to investigate the seroepidemiology of *T. gondii* infection in Huicholes in Mexico. Results indicate that Huicholes have one of the highest seroprevalences of *T. gondii* infection reported in the region. The seroprevalence found in Huicholes (33.2%) is higher than the mean (23.8%) seroprevalence of *T. gondii* infection reported in the general population in rural areas in Durango State [18]. In addition, the seroprevalence in Huicholes is higher than the 6.1% seroprevalence of *T. gondii* infection reported in urban general population in the capital Durango City [19]. With respect to other ethnic groups in the region, the seroprevalence found in Huicholes is comparable with the 30.3% seroprevalence of *T. gondii* infection reported in Mennonites [9] but is higher than the 22.4% seroprevalence reported in Tepehuanos [10]. Huicholes and Tepehuanos live in the same mountains (Sierra Madre Occidental), however, Huicholes live in more remote places deeper into the mountainous region than Tepehuanos. It is known that the seroprevalence of *T. gondii* infection varies depending on the climate conditions in the communities, i.e., a low seroprevalence in dry and hot climate [14], and high seroprevalence in humid regions [12]. Huicholes communities are located in a lower region on the mountains with warmer and more humid climate than the one of the Tepehuanos settlements. However, difference in the seroprevalences among Huicholes and Tepehuanos should be interpreted with care because of an age limitation of the comparison: the mean age in Tepehuanos (31.03 ± 16.71 years old) was lower than the one (37.98 ± 15.80 years old) in Huicholes.

*Toxoplasma* exposure has been linked to low socioeconomic status [20,21], and such characteristics might have contributed to the increased seroprevalence of *T. gondii* infection in Huicholes. The seroprevalence of *T. gondii* infection in Huicholes with low socioeconomic status (34.3%) was higher than in those with medium socioeconomic status (15.4%). However, such increase was not statistically significant because of the limited number (n = 12) of subjects with medium socioeconomic status in the comparison. In the present study women outnumber men. The higher number of women than men in the study may be due to a number of factors including refusal of some men to participate, and more migration and violent deaths in men than in women in the study region. However, the imbalance in sexes in this study is unlikely to affect the seroprevalence rate since seroprevalence of *T. gondii* infection has been found similar in men and in women of general populations in rural [18] and urban [19] Durango, Mexico.

Multivariate analysis of the socio-demographic and behavioral characteristics of the Huicholes allowed us to identify that consumption of turkey meat was positively associated with *T. gondii* exposure in Huicholes. In a previous study in the general population in rural Durango, consumption of turkey meat was also associated with *T. gondii* exposure [18]. In addition, in a study in pregnant women in the urban capital Durango City, consumption of turkey meat was associated with *T. gondii* infection too [22]. Turkey meat is a potential source for infection with *T. gondii*[23]. Experimental *T. gondii* oocyst infections in turkeys have shown the parasite spreading over the whole organism as determined by polymerase chain reaction [24]. In a previous study in birds in Durango, no serological evidence of *T. gondii* infection in 16 turkeys (*Meleagris gallopavo*) was found [25]. However, the number of studied turkeys was too small to exclude *T. gondii* infections in turkeys in Durango. Turkey meat is frequently cooked in big pieces; therefore, it is likely that this meat remains undercooked in some deep areas.

Seroprevalence of *T. gondii* infection usually increases with age in our region [18,19,26]. In the present survey, seroprevalence tended to increase with age; however, such increase did not reach statistical significance (*P* = 0.09). It is likely that the small sample size of the oldest Huicholes subgroup prevented us obtaining a statistically significant difference.

Remarkably, in the present study we found an association of *T. gondii* exposure with the presence of dizziness and memory impairment in Huicholes. This finding may indicate a causal association of infection with *T. gondii* and central nervous system illness in Huicholes. The association of *T. gondii* exposure with dizziness and memory impairment was also found in a recent study in migrant agricultural workers in Durango [21]. The association of memory impairment and *T. gondii* exposure was also assessed in other ethnic groups in Durango including Mennonites [9] and Tepehuanos [10]; however, no association was found. The association of dizziness and *T. gondii* exposure was not assessed in Mennonites [9] and Tepehuanos [10]. We are not aware of further reports on the association of dizziness with *T. gondii* infection. On the other hand, the association of memory impairment with *T. gondii* infection found in the present study agrees with previous reports [27,28]. In a previous study in gardeners in Durango City, *T. gondii* seropositivity was associated with memory impairment [27]. In a recent study in seniors in Germany, researchers found that *T. gondii* seropositivity was associated with a reduction of about 35% in working memory, a lower performance in verbal memory, and a decreased quality of life [28]. A number of reports indicate that *T. gondii* infection may lead to neurological and behavioral changes. Experiments in adult mice have shown that infections with *T. gondii* cause neurological and behavioral abnormalities secondary to inflammation and loss of brain parenchyma [29]. In addition, chronic infections with *T. gondii* in mice can damage the spatial learning and memory capability [30]. The behavioral alterations associated with *T. gondii* infection in humans and animals were recently reviewed by Flegr [31] and Webster *et al*., [32]. Intriguingly, latent *T. gondii* infection was associated with improvements in cognitive control processes in young healthy humans [33]. However, this effect might perhaps not be found or even reversed in old age. Beste *et al*. [34] found that latent *T. gondii* infection leads to deficits in goal-directed behavior in otherwise healthy elderly individuals.

Anti-*T. gondii* IgM antibodies were present in a high number of anti-*T. gondii* IgG positive Huicholes. Such high frequency of IgM positive results was unexpected and should be interpreted with care since IgM ELISA kits have a high rate of false positive results [35]. Therefore, discrimination between recent and latent *T. gondii* infections is not accurately obtained by ELISA. Other methods such as IgG avidity [36,37] may aid in such discrimination.

The present study has some limitations, including the sampling method and the small sample size of elderly Huicholes. We were unable to perform random sampling because participation of Huicholes in the study depended to a large extent on the permission of the Huicholes leaders. Huicholes leaders asked their people to participate and only Huicholes who attended the invitation were sampled. A small number of elders attended the invitation.

## Conclusion

Our results demonstrate serological evidence of *T. gondii* exposure in Huicholes and *Toxoplasma* may be impacting their health. This is the first report of *T. gondii* infection in Huicholes, and results should be useful for the optimal design of preventive measures against *T. gondii* infection.

## Competing interests

The authors declare that they have no competing interest.

## Authors’ contributions

CAE conceived and designed the study protocol, participated in the coordination and management of the study, performed the laboratory tests and the data analysis and wrote the manuscript. SJPV, LOBS, FJIRA, OAGG and FCR obtained blood samples, submitted the questionnaires and performed the data analysis. JHT, IBG, OL, LFSA and ERS performed the data analysis, and wrote the manuscript. All authors read and approved the final version of the manuscript.
